# Supplementary material for: Synaptic proteomics reveal distinct molecular signatures of cognitive change and C9ORF72 repeat expansion in the human ALS cortex
Source: Acta Neuropathol Commun. 2022 Oct 29;10:156. doi: 10.1186/s40478-022-01455-z (PMC9618182; doi:10.1186/s40478-022-01455-z)
Supplement: Supplementary file 2 — Additional file 2. All Supplementary Figures and legends. [file 40478_2022_1455_MOESM2_ESM.docx]

**Synaptic proteomics reveal distinct molecular signatures of cognitive change and *C9ORF72* repeat expansion in the human ALS cortex**

Zsofia I. Laszlo^1^, Nicole Hindley^1^, Anna Sanchez Avila^1,2^, Rachel A. Kline^2^,^3^, Samantha L. Eaton^3^, Douglas J. Lamont^4^, Colin Smith^2,5^, Tara L. Spires-Jones^2,6^, Thomas M. Wishart^2,3^, Christopher M. Henstridge^1,2^*

**Supplementary Figures 1-6**


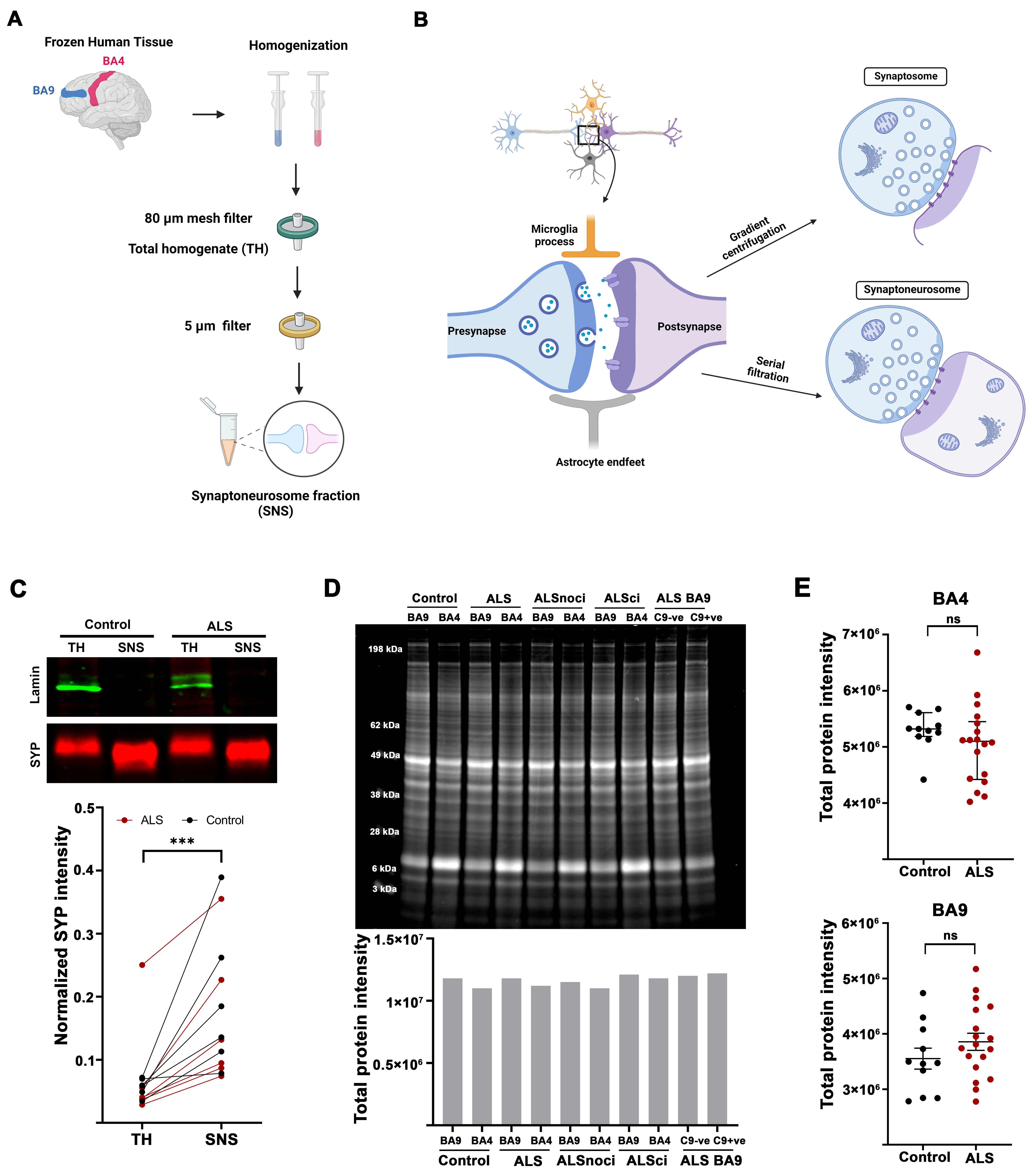


**Supplementary Figure 1. Graphical summary of experimental plan and synaptic preparations.**

**A**. Graphical representation of the synapse enrichment protocol. **B**. Schematic showing the composition of a synaptoneurosome preparation. Our serial filtration approach retains re-sealed pre and postsynaptic compartments, plus their constituent membrane and cytosolic proteins. Also, small organelles such as mitochondria and RNA-processing machinery will be kept. Finally, tighly-bound glial processes will also be extracted within these samples. This is different to the synaptosome approach which inherently loses many postsynaptic proteins. **C**. Western blots from control and ALS samples confirm exclusion of nuclear protein (lamin) and enrichment of synaptic protein (synaptophysin) in synaptoneurosome (SNS) preps. Paired t-test, ***p<0.0001. **D.** Total protein Coomassie stain of a gel containing 5ug of each group sample shows similar protein intensity and banding patterns. **E.** Quantification reveals no difference in total protein levels between individual control and ALS samples in both brain regions. BA4: Mann-Whitney test, p= 0.1015, BA9: Unpaired t-test p=0.2293. Graphs show individual cases per group (dots) and median ± IQR or mean ± SEM.


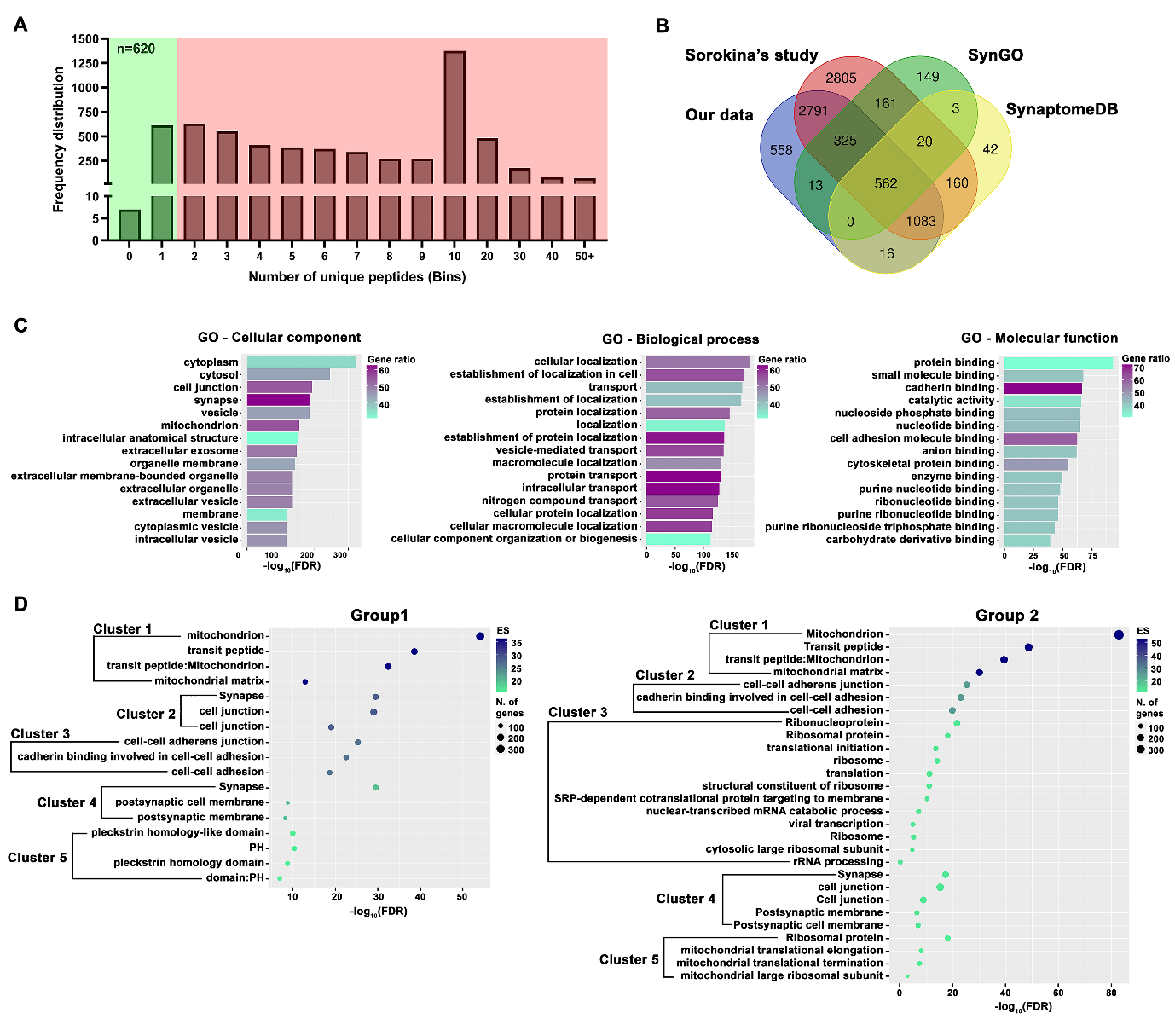


**Supplementary Figure 2: Further enrichment analysis of our synaptic samples.**

**A.** All protein IDs derived from fewer than two unique peptides (n=620) were excluded from further analysis. **B.** Filtered, final dataset was aligned with 3 different synaptic databases, and showed ~90% overlap, thereby validating the composition of our synaptic fractions. **C.** Enrichment analysis was performed using g:Profiler. Gene Ontology (GO) clusters were plotted using the top 15 biological terms, ranked by -log10(FDR) and coloured by gene ratio (gene number/term size). **D.** Functional annotation clustering was performed using DAVID Functional Annotation Bioinformatics Microarray Analysis software. Our large dataset was halved using a random group generator (DAVID only accepts inputs of 3000 IDs) and each group was uploaded into DAVID and analysed. Both plots represent the top 5 clusters with different biological terms, plotted by -log10(FDR) and gene count (dot size), coloured based on the DAVID enrichment score per cluster.


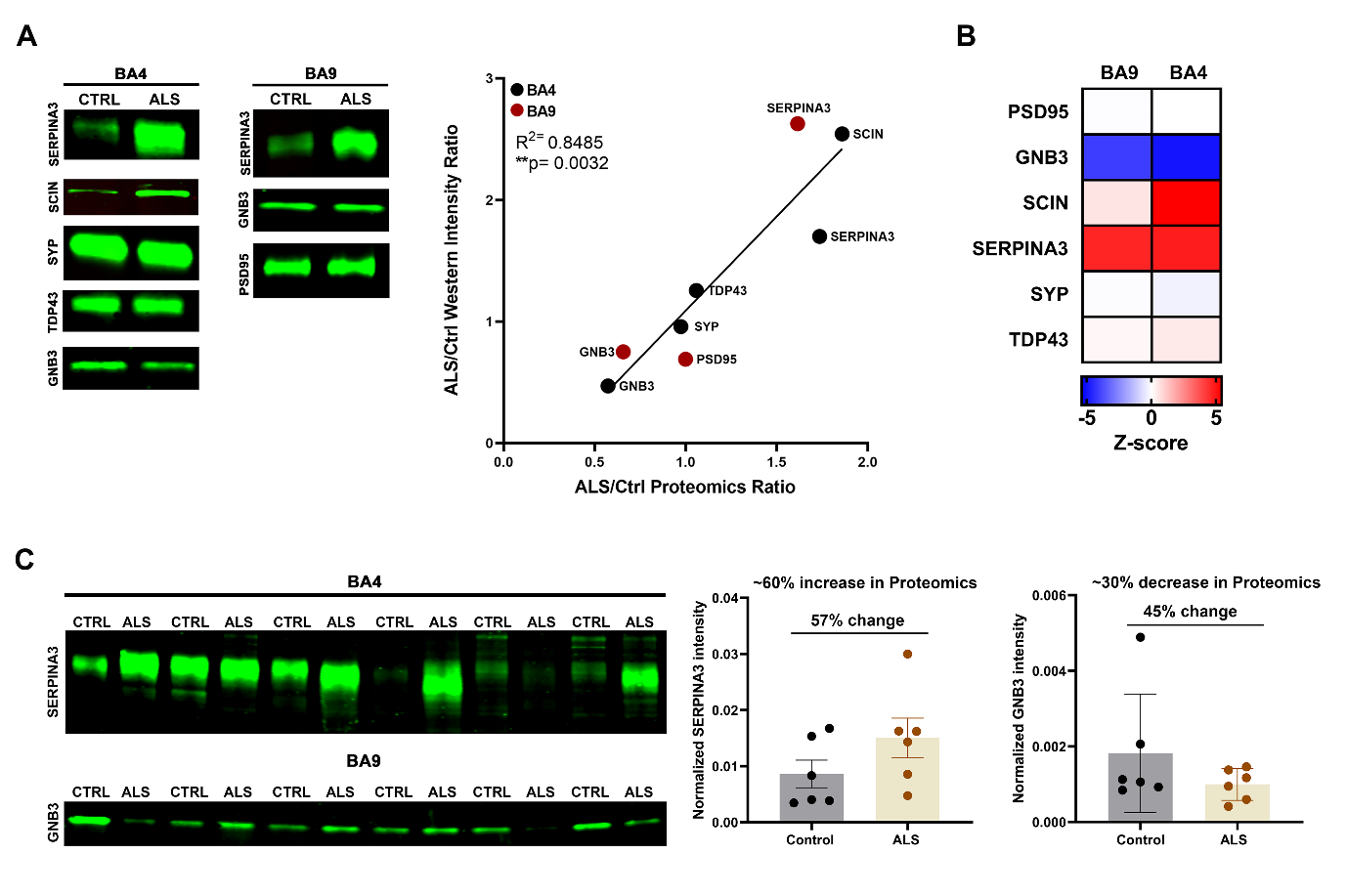


**Supplementary Figure 3: Comparison of proteomics data with western blot analyses.**

**A.** Representative western blot images using BA4 and BA9 pooled samples shows significant correlation with proteomics results. Control normalized proteomics intensity values (corrected by 1/median) were plotted against control normalized western blot intensity values. Pearson’s correlation, R^2^=0.8485, **p=0.0032. **B.** Heatmap representation of proteins in **(A)**. **C**. Western blot images show SERPINA3 and GNB3 expression in individual cases from different brain areas. Although the quantification shows no significant difference between Ctrl and ALS groups, there is a coherence with the proteomics data. SERPINA3: Unpaired t-test, p=0.1681; GNB3: Mann-Whitney test, p=0.5887. Graphs show mean ± SEM or median ± IQR.


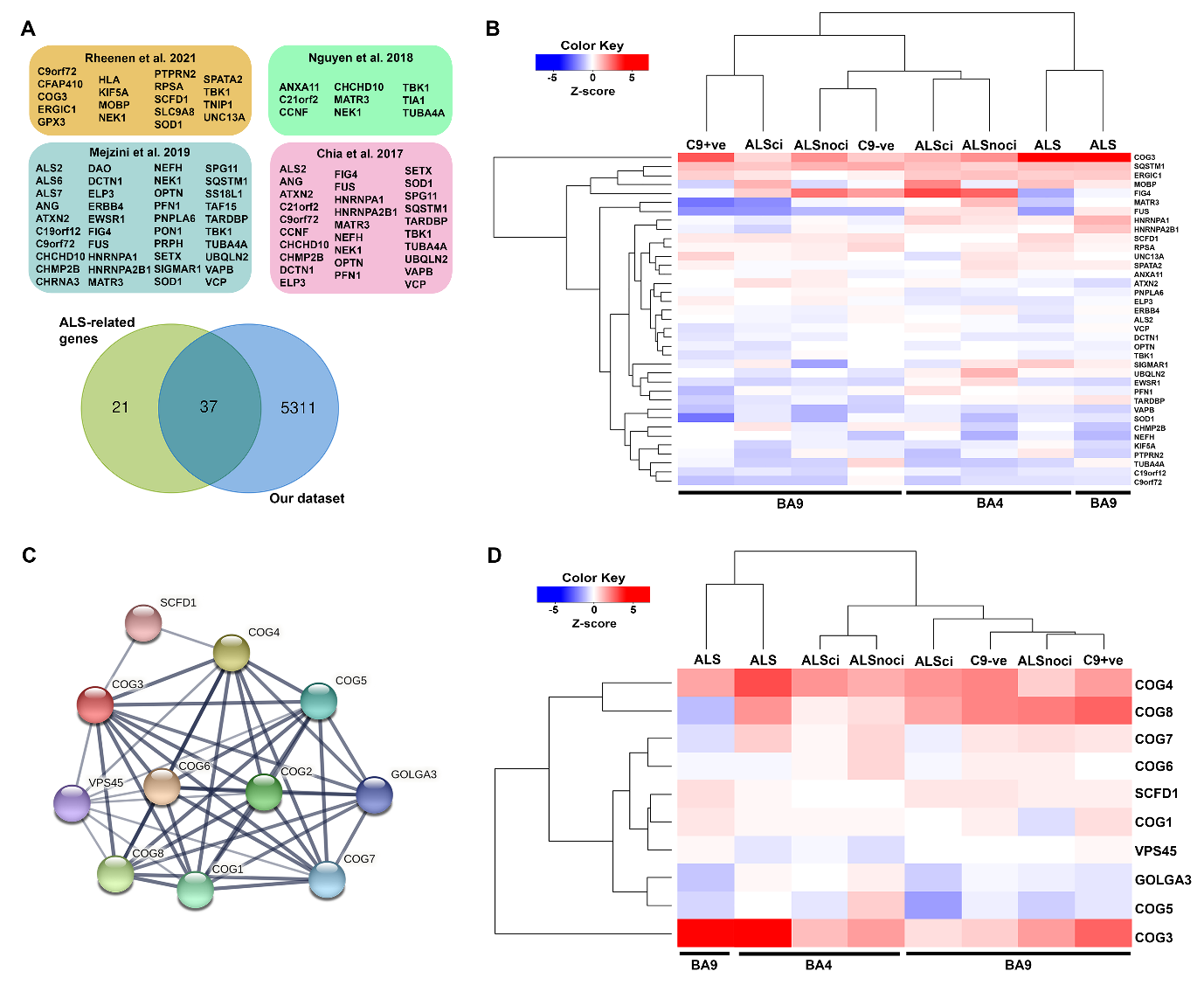


**Supplementary Figure 4. Expression profile of ALS-related genes.**

**A**. 58 ALS-related genes were collated from the 4 highlighted references. **B**. Heatmap showing the differences in expression of the protein product from the 37 ALS-related genes found in our dataset. **C**. Interaction map of the most upregulated gene, COG3. Diagram was made using the STRING database, edges indicate that the proteins are part of a physical complex, meanwhile the line thickness indicates the strength of supporting data. **D.** Heatmap showing the protein expression profile of the COG3-based network highlighted in **C**. Heatmaps was made using hierarchical clustering with Euclidean distance.


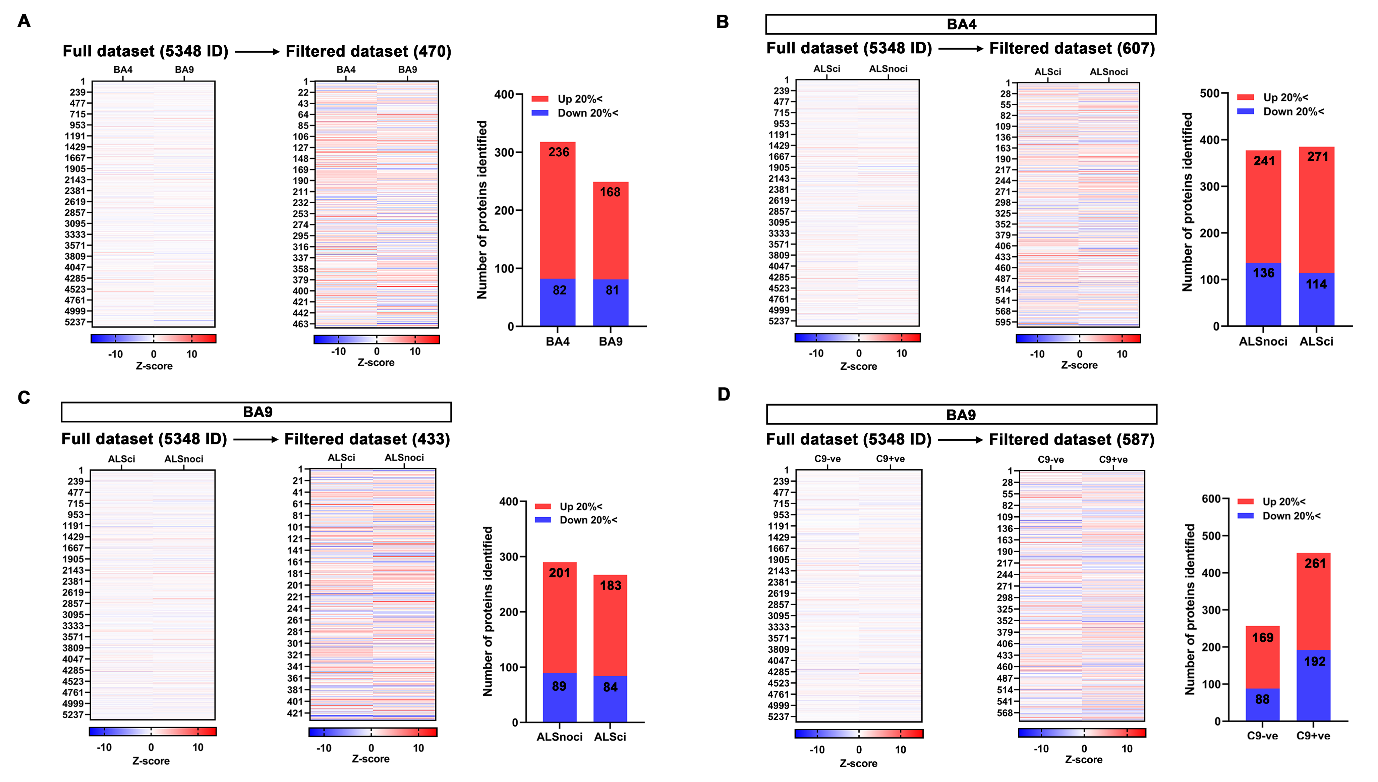


**Supplementary Figure 5: Data filtration to identify proteins for further analysis.**

**A-D.** Heatmaps show the filtration process of the data in each experimental comparison. The first heatmap represents the expression change of all 5348 proteins versus control samples, and the second represents the proteins that have altered expression (± 20%) versus control. Bar graphs represent the number of up- and downregulated proteins in each group versus control. Data was stratified by brain region (**A**), cognitive performance (**B** and **C**) and genetic status (**D**).

**Supplementary Figure 6. Analysing protein change at single synapse resolution.**

**A.** Example micrographs of synaptophysin (SYP) and synaptopodin (SYNPO) staining of post-mortem human tissue sections using array tomography. Single channel images suggest increased density of SYNPO puncta in c9or72-RE+ve tissue. **B.** Synapses consisting of presynaptic SYP and postsynaptic SYNPO were consistent throughout the neuropil. This example was obtained following segmentation and 3D rendering of array tomography images which were used for quantification, using the free imaging tool Paraview (paraview.org). **C.** Graphs show the density of SYP or SYNPO in each group using 5 cases per group. SYNPO shows a similar change in C9ORF72-RE+ve samples that ratiometric proteomics data showed. Groups were compared with one-way ANOVA using Tukey’s multiple comparison test, no significant difference were found. Graphs show mean±SEM.
