# Supplementary material for: Synaptic proteomics reveal distinct molecular signatures of cognitive change and C9ORF72 repeat expansion in the human ALS cortex
Source: Acta Neuropathol Commun. 2022 Oct 29;10:156. doi: 10.1186/s40478-022-01455-z (PMC9618182; doi:10.1186/s40478-022-01455-z)
Supplement: Supplementary file 5 — Additional file 5. Appendix 1, example images from whole western blots used in this manuscript [file 40478_2022_1455_MOESM5_ESM.pdf]

Additional information

Synaptic proteomics reveal distinct molecular signatures of cognitive change and C9orf72 repeat expansion in the human ALS cortex

Zsofia I. Laszlo, Nicole Hindley, Anna Sanchez Avila, Rachel A. Kline, Sam Eaton, Douglas Lamont, Colin Smith, Tara L. Spires-Jones, Tom Wishart, Christopher M. Henstridge

Method validation blots for Supplementary Figure 1/C.

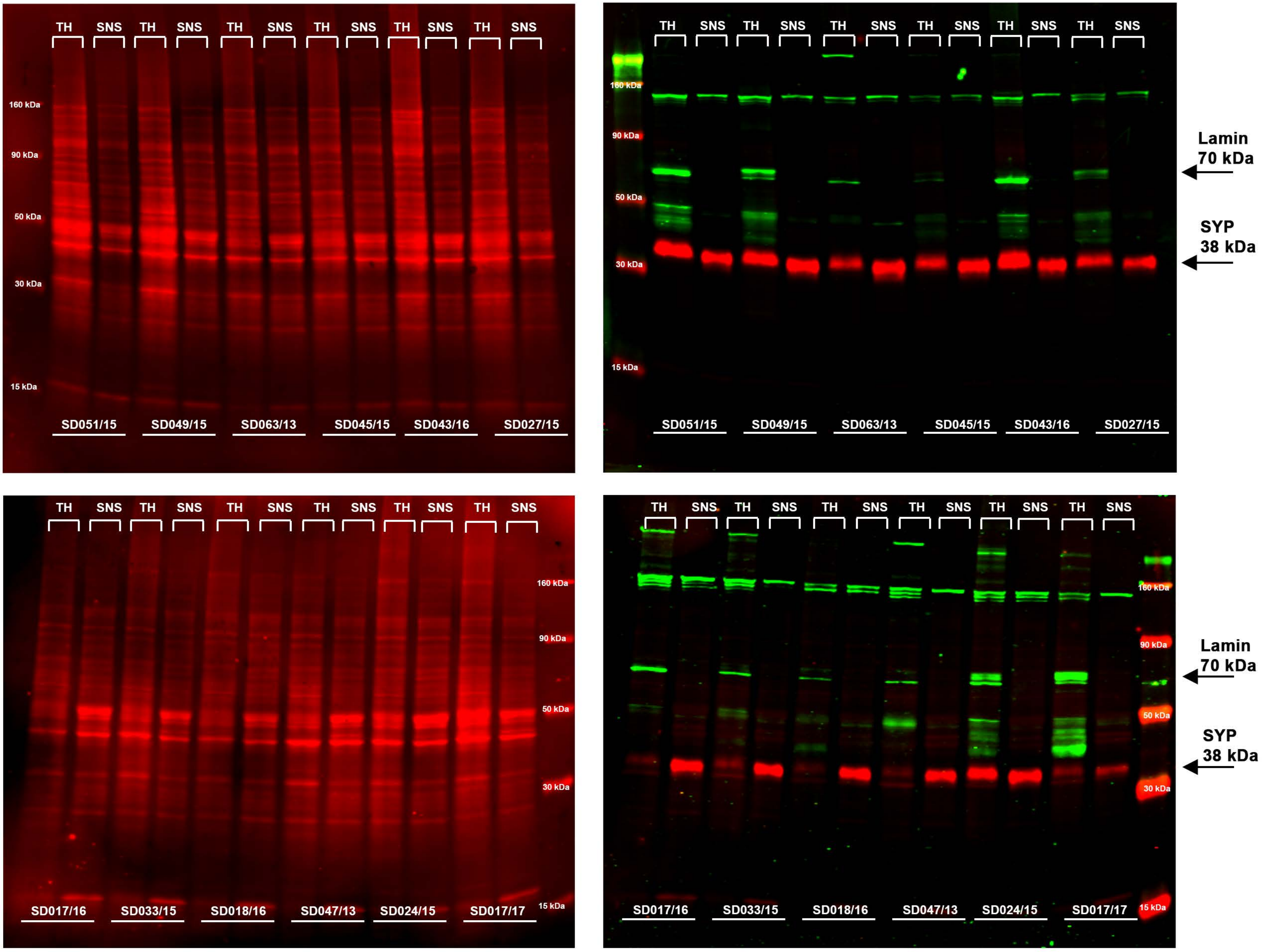

Total protein stain for Supplementary Figure 1/D.

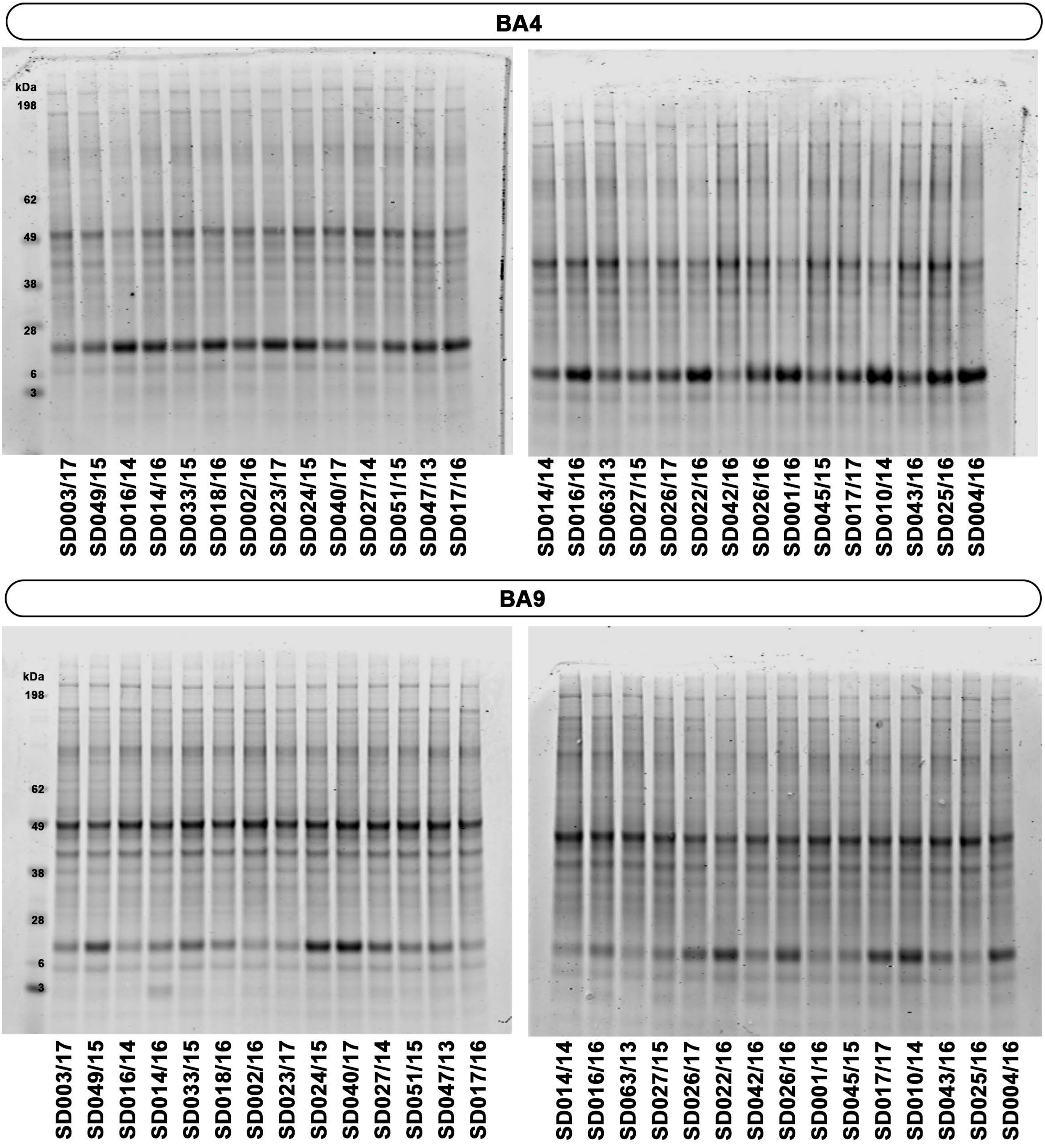

Proteomics validation for Supplementary Figure 3/A.

BA4

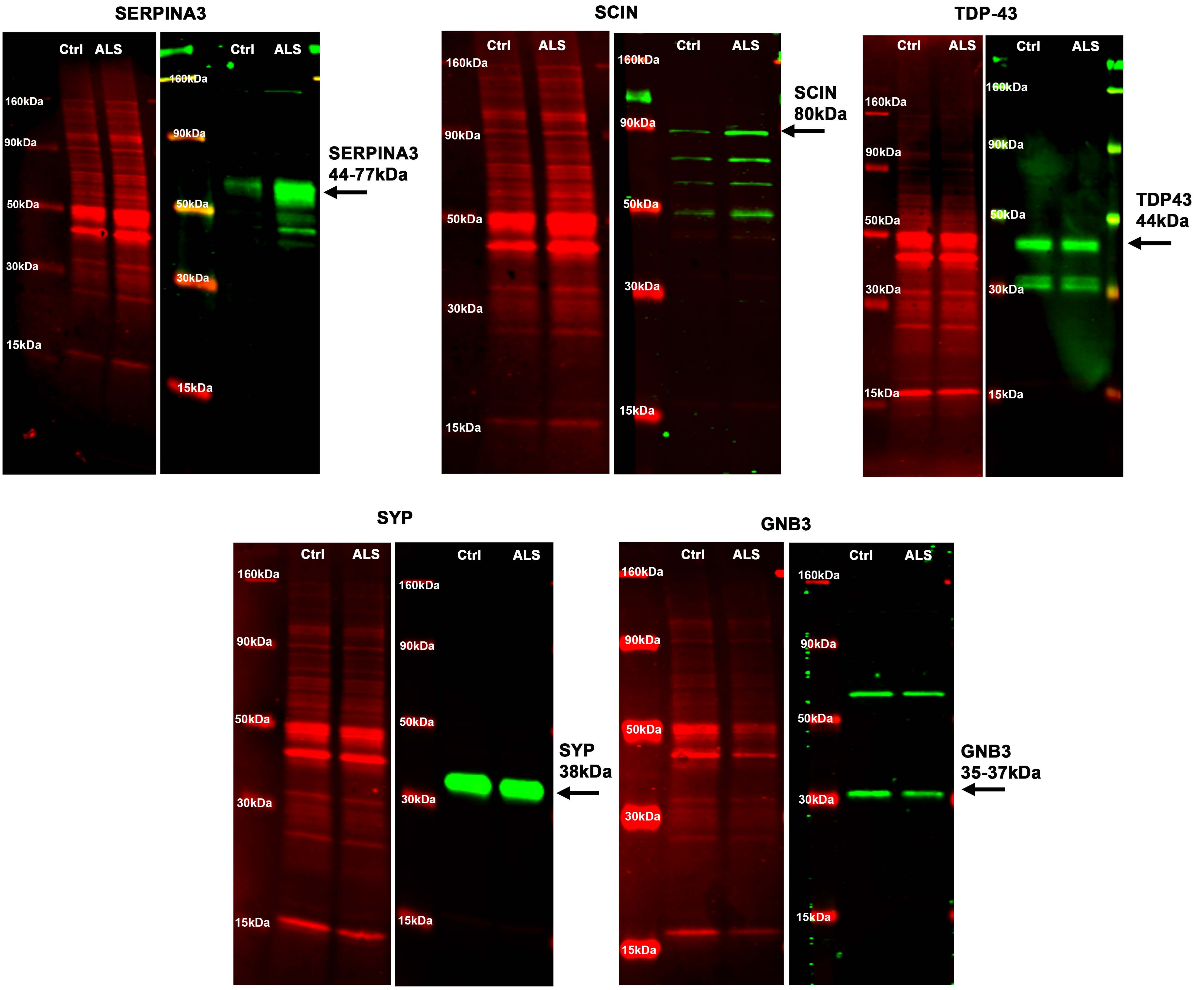

BA9

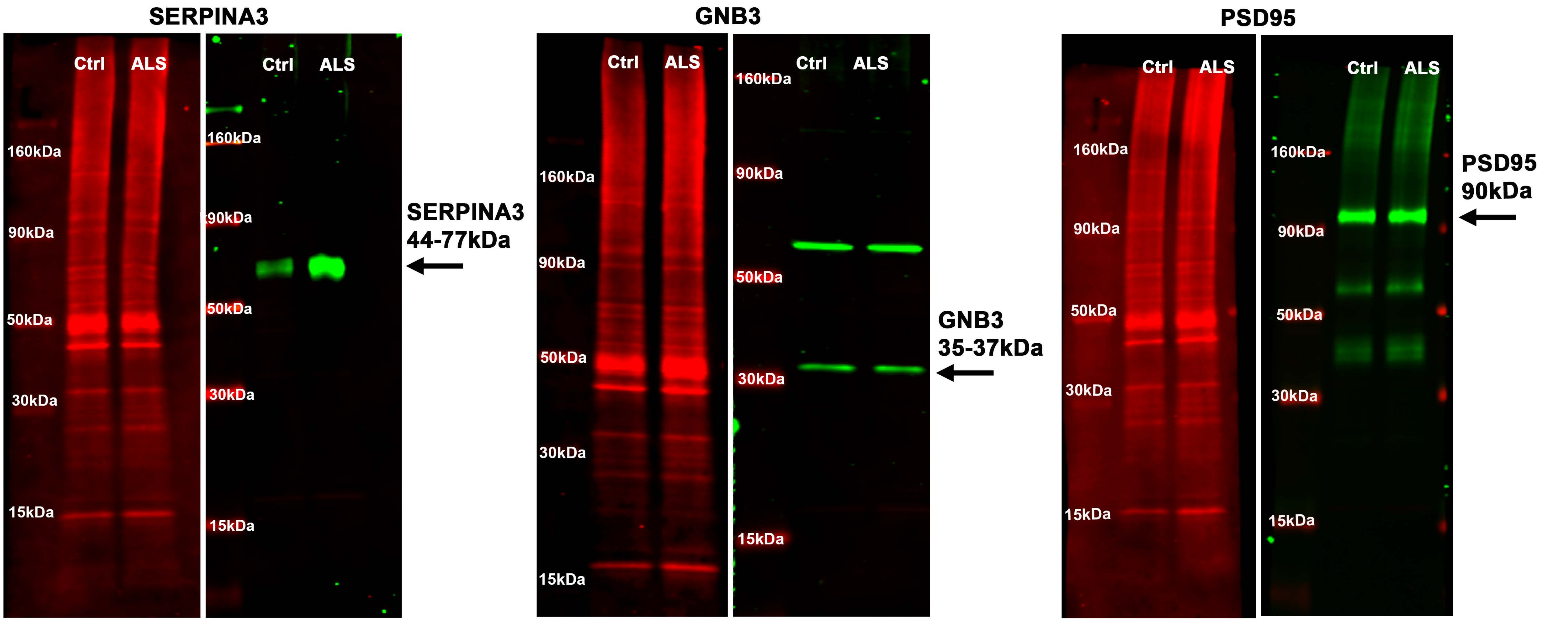

Western blots using individual samples for Supplementary Figure 3/C.

SERPINA3 - BA4

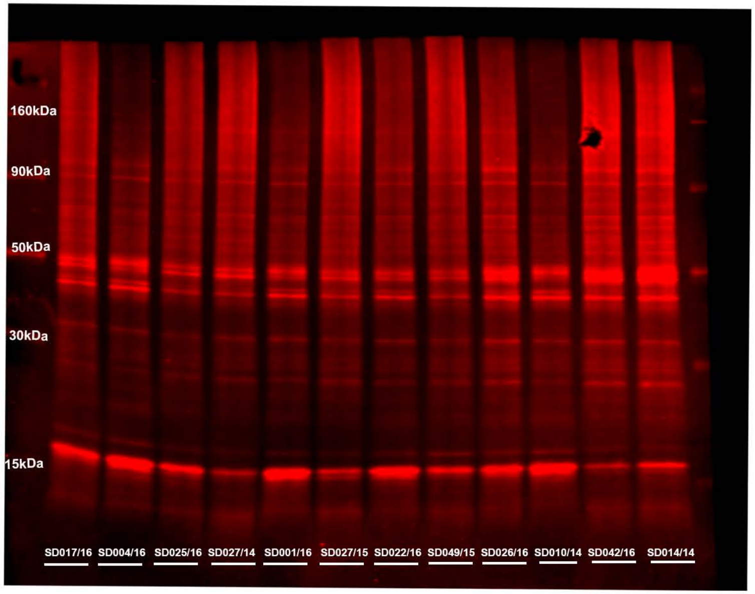

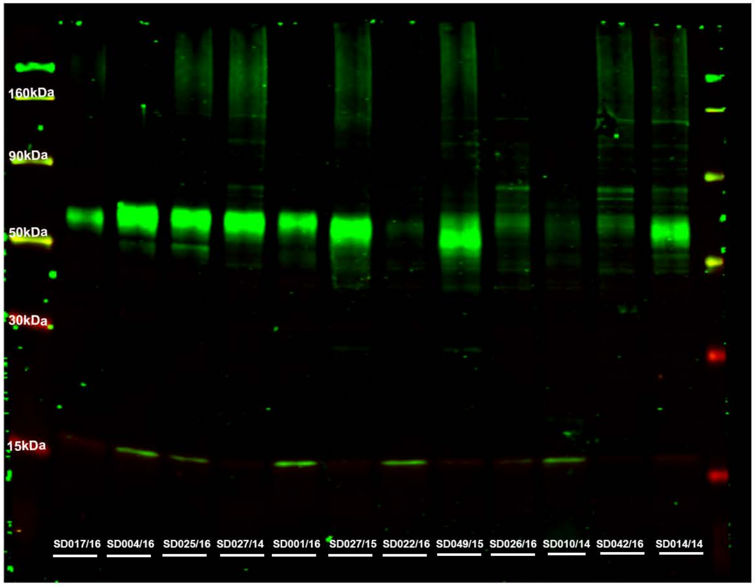

SERPINA3
44-77 kDa

GNB3 - BA9

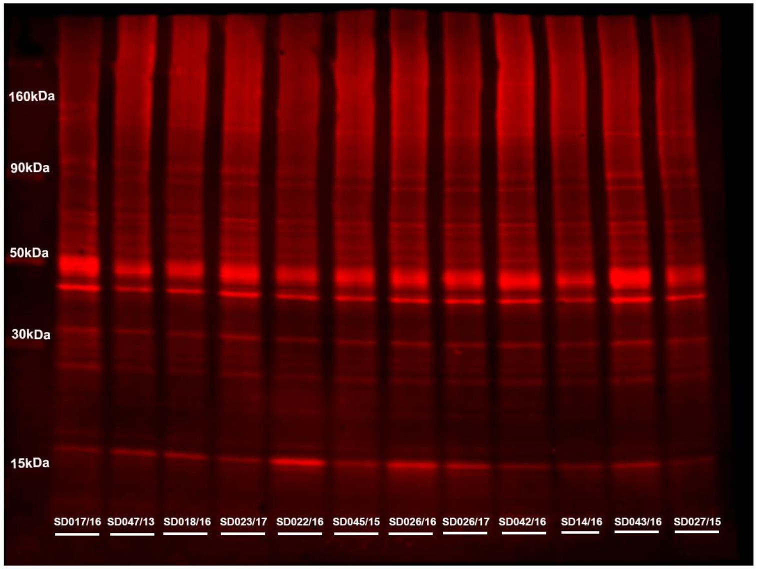

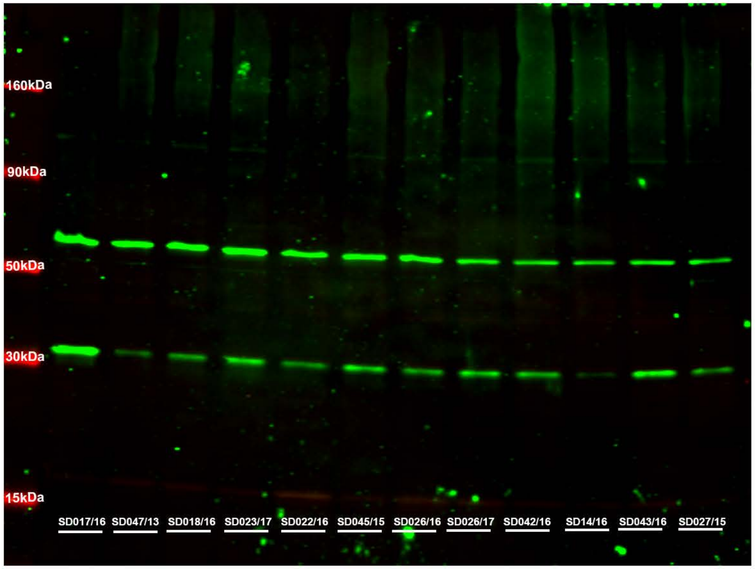

GNB3
35-37kDa
